# Identification of inflammatory biomarkers in IgA nephropathy using the NanoString technology: a validation study in Caucasians

**DOI:** 10.1007/s00011-023-01848-3

**Published:** 2024-01-31

**Authors:** Laurence Gaumond, Caroline Lamarche, Stéphanie Beauchemin, Nathalie Henley, Naoual Elftouh, Casimiro Gerarduzzi, Louis-Philippe Laurin

**Affiliations:** 1 Division of Nephrology, Maisonneuve-Rosemont Hospital, 5415 Boulevard de l’Assomption, Montreal, QC H1T 2M4 Canada; 2 Research Center, Maisonneuve-Rosemont Hospital, Montreal, QC Canada; 3 Department of Medicine, University of Montreal, Montreal, QC Canada

**Keywords:** IgA nephropathy, Glomerulonephritis, NanoString technology, Biomarkers, Inflammation, Proteinuria, End-stage kidney disease, Prognosis

## Abstract

**Objective and design:**

Immunoglobulin A nephropathy (IgAN) is a kidney disease characterized by the accumulation of IgA deposits in the glomeruli of the kidney, leading to inflammation and damage to the kidney. The inflammatory markers involved in IgAN remain to be defined. Gene expression analysis platforms, such as the NanoString nCounter system, are promising screening and diagnostic tools, especially in oncology. Still, their role as a diagnostic and prognostic tool in IgAN remains scarce. In this study, we aimed to validate the use of NanoString technology to identify potential inflammatory biomarkers involved in the progression of IgAN.

**Subjects:**

A total of 30 patients with biopsy-proven IgAN and 7 cases of antineutrophil cytoplasmic antibody (ANCA)-associated pauci-immune glomerulonephritis were included for gene expression measurement. For the immunofluorescence validation experiments, a total of 6 IgAN patients and 3 controls were included.

**Methods:**

Total RNA was extracted from formalin-fixed paraffin-embedded kidney biopsy specimens, and a customized 48-plex human gene CodeSet was used to study 29 genes implicated in different biological pathways. Comparisons in gene expression were made between IgAN and ANCA-associated pauci-immune glomerulonephritis patients to delineate an expression profile specific to IgAN. Gene expression was compared between patients with low and moderate risk of progression. Genes for which RNA expression was associated with disease progression were analyzed for protein expression by immunofluorescence and compared with controls.

**Results:**

IgAN patients had a distinct gene expression profile with decreased expression in genes *IL-6, INFG,* and *C1QB* compared to ANCA patients. *C3* and *TNFRSF1B* were identified as potential biomarkers for IgAN progression in patients early in their disease course. Protein expression for those 2 candidate genes was upregulated in IgAN patients compared to controls. Expression of genes implicated in fibrosis (*PTEN*, *CASPASE 3*, *TGM2*, *TGFB1*, *IL2*, and *TNFRSF1B*) was more pronounced in IgAN patients with severe fibrosis compared to those with none.

**Conclusions:**

Our findings validate our NanoString mRNA profiling by examining protein expression levels of two candidate genes, *C3* and *TNFRSF1B*, in IgAN patients and controls. We also identified several upregulated mRNA transcripts implicated in the development of fibrosis that may be considered fibrotic markers within IgAN patients.

## Introduction

Immunoglobulin A nephropathy (IgAN) is a complicated autoimmune condition where immunoglobulin A (IgA) is deposited in the kidney’s glomeruli, causing inflammation and ultimately leading to kidney failure [1]. IgAN is a leading cause of primary glomerulonephritis and chronic kidney disease [2]. Several clinical factors have been identified over the last decades to help predict disease progression, such as sustained proteinuria over a gram a day, baseline estimated glomerular filtration rate (eGFR), high blood pressure, and histopathologic findings from the Oxford classification [3]. In IgAN, the search for inflammatory biomarkers that can be utilized to track therapy effectiveness and foretell disease development remains unfinished [4]. No one biomarker can give a comprehensive picture of IgAN, so several biomarkers may be required to diagnose and treat the disease correctly. Although a number of clinical indicators have been found to help predict disease progression, specific biomarkers involved in the underlying inflammatory processes are not integrated into any prediction tool. Therefore, further research is needed to validate the clinical utility of inflammatory biomarker panels in IgAN.

State-of-the-art platforms for gene expression analysis, such as the NanoString nCounter system, are promising screening and diagnostic tools, especially in oncology. This innovative process, which directly measures individual micro ribonucleic acid (mRNA) transcripts without enzymatic steps, stands out from previous methods by its high sensitivity, precision, and efficiency [5]. Specifically, the NanoString nCounter system uses a digital molecular barcoding technology, which allows for high-throughput and sensitive detection of specific RNA molecules in a sample without amplification. It is often used in research to study gene expression, biomarker discovery, and pathway analysis. Increasingly used in several clinical settings, this technology could help identify biomarkers playing a role in various inflammatory pathologic processes.

This pilot study successfully validates the application of NanoString technology in identifying potential inflammatory targets related to different stages of IgAN disease progression. Our results revealed a distinctive gene expression profile in IgAN patients. Notably, we identified *C3* and *TNFRSF1B* as promising biomarkers for monitoring IgAN progression, with their protein expression being significantly upregulated in IgAN patients compared to controls. Additionally, our investigation uncovered heightened expression of genes associated with fibrosis in IgAN patients with severe fibrosis compared to those without fibrosis. This finding suggests the potential utility of these mRNA transcripts as fibrotic markers for IgAN patients. The successful validation of NanoString mRNA profiling and the identification of relevant inflammatory and fibrotic markers may pave the way for improved diagnostic and prognostic tools in IgAN, enhancing our understanding of the disease’s underlying inflammatory processes.

## Methods

### Study design and population

All patients with biopsy-proven IgAN and antineutrophil cytoplasmic antibody (ANCA)-associated pauci-immune glomerulonephritis between 2005 and 2021 diagnosed at Maisonneuve-Rosemont Hospital, a single tertiary care hospital in Montreal, were considered for this retrospective study. Only patients with formalin-fixed paraffin-embedded (FFPE) biopsy samples available for gene expression analysis were included. Patients with known secondary causes of IgAN were excluded. For the immunofluorescence experiments, controls with no glomerular disease (after pathology review) were recruited among patients who underwent a nephrectomy in the context of a kidney lesion. All subjects provided written informed consent. This study was approved by our institution’s Research Ethics Committee (No MP-12-2014-525) in agreement with the Declaration of Helsinki.

### Data collection

Clinical data were collected by reviewing medical records, including demographics, kidney presentation, comorbidities, history of familial kidney disease, and medications. Specifically, serum creatinine, eGFR, and proteinuria were noted at the time of the biopsy, at 6 months from biopsy time and then annually to the last available follow-up visit and/or initiation of renal replacement therapy. The Oxford MEST-C score [6] was retrieved from the biopsy report. Baseline eGFR was estimated using the Chronic Kidney Disease Epidemiology Collaboration equation [7]. Urinary protein excretion was estimated using either total 24-h proteinuria (in g/day), spot urinary protein–creatinine ratio (in mg/mmol), or spot urinary albumin–creatinine ratio (in mg/mmol). For IgAN, the risk of a 50% decline in eGFR or progression to end-stage kidney disease (ESKD) 5 years after kidney biopsy was calculated using the New International Risk-Prediction Tool in IgAN [8], a prediction model that includes baseline clinical and pathological characteristics, demographics, and medication use. The studied primary outcomes were ESKD (eGFR < 15 ml/min/1.73m^2^, dialysis or kidney transplantation) or death.

### Gene expression measurement

For our NanoString analysis, a total of 30 biopsy-confirmed IgA and 7 ANCA-positive pauci-immune glomerulonephritis patients were used. Total RNA from FFPE kidney biopsy specimens from patients with IgAN and ANCA-positive pauci-immune glomerulonephritis (control group) were extracted and purified using RNeasy FFPE kit (Qiagen) according to the manufacturer’s protocol. A total of 100 nanograms of RNA from each sample was hybridized to a customized 48-plex human gene CodeSet and processed on the nCounter platform. Purified RNA samples were analyzed using the nCounter gene expression system from NanoString Technology. A total of 29 genes implicated in different biological pathways were studied; those genes have been selected for their role in complement activation (*C2*, *C3*, *C1QA*, *C1QB*), apoptosis (*BAX*, *BAD*, *Caspase 3*), cell activation, and/or leucocytes stimulation (*AGTR1*, *AGTR2*, *CXCL1*, *CD89S*, *ICAM1*, *MIF*, *PTEN*, *CD71*, *TGM2*, *TNFR1B*, *TNFRSF1B*, *TNFSF13*, *IL-2*, *IL-4*, *IL-5*, *IL-6*, *IL-8*, *IL-10*, *IL-13*, *IFN-γ*, *TGF-β1*, *TNF*). Housekeeping gene (*ACTB*, *GAPDH*, *HPRT1*, *LDHA*) normalization was performed to adjust counts of all probes relative to a probe that is not expected to vary between samples; the factor should be in the range between 0.1 and 10. All normalization procedures were performed according to the manufacturer’s protocol.

Data collection for gene expression was carried out in the nCounter Digital Analyzer. At the highest standard resolution, 555–1155 fields of view were collected per flow cell using a microscope objective and a CCD camera, yielding data of hundreds of thousands of target molecule counts. Every RNA target is identified by the color code generated by the ordered fluorescent segments present on the reporter probe. The expression level of each gene is determined by scoring the number of times its corresponding color code is detected. Data were imported into nSolver Analysis Software for downstream analysis.

Comparisons in gene expression were made between IgAN patients and those with another inflammatory glomerulonephritis (ANCA-associated pauci-immune glomerulonephritis) to depict an expression profile specific to IgAN. Subsequently, among individuals with IgAN, gene expression was compared between patients with a low chance of progression to ESKD (< 10%) and those at mild to moderate risk of progression (10–20%) with relatively minimal fibrosis on kidney biopsy. Genes for which RNA expression was associated with disease progression were analyzed in 6 patients for protein expression by immunofluorescence and compared with controls.

### Histology

The same nephropathologist reviewed histologic slides for all patients with IgAN to provide MEST-C score: mesangial hypercellularity (with M0 and M1 corresponding to ≤ 50% and  > 50% of glomeruli with hypercellularity, respectively), segmental glomerulosclerosis (with S0 if absent and S1 if present), endocapillary hypercellularity (with E0 if absent and E1 if present), tubular atrophy/interstitial fibrosis (with T0, T1, and T2 corresponding to ≤ 25%, 26%–50%, and > 50% of cortical area involvement, respectively), and cellular/fibro cellular crescents (with C0, C1, and C2 corresponding to their absence, presence in ≥1 and < 25% of glomeruli, and presence in ≥25% of the glomeruli, respectively). ANCA-associated pauci-immune glomerulonephritis was also graded based on its degree of tubular atrophy/interstitial fibrosis.

### Immunofluorescence

The kidneys were fixed in 10% formaldehyde, dehydrated, and embedded in raffin. The tissue was sectioned (5 µM) and was subjected to antigen retrieval in citrate solution at pH 6. The sections were blocked with anti-donkey serum 5% and labeled with anti-Complement C3 (Catalog #PA1-29715 from Life Technologies/ThermoFisher) and TNFRSF1B (Catalog #MA5-31661 from Life Technologies/ThermoFisher). The slides were subsequently exposed to donkey anti-rabbit AF647-conjugated (1:400, Catalog # 711 605 152 Jackson ImmunoResearch Laboratories), donkey anti-mouse AF568 (Catalog # A10037 Life Technologies/ThermoFisher), and donkey anti-goat AF488 (Catalog # A11055 Life Technologies/ThermoFisher). Fluoroshield with DAPI (Millipore-Sigma) was used for nuclear staining and mounting. Slides were imaged using a Zeiss AxioObserver.Z1 inverted microscope coupled to an X-Cite 120LED Boost High-Power LED illumination system. Images for quantitative analysis were captured with a 20X objective and the number of positive cells was determined as the average of positive cells in at least 8 fields per kidney section.

### Statistical analysis

Baseline characteristics, clinical and pathological parameters, and gene expression were compared between patients with IgAN and those with ANCA-associated pauci-immune glomerulonephritis (controls). Among patients with IgAN, gene expression was compared between progression score groups (< 10%, 10–20%, 21–40%, and > 40%), and more specifically between patients with scores < 10% and those with 10–20% to delineate markers of early disease. Wilcoxon or *t*-tests for continuous variables and the *χ*^2^ test or the Fisher exact tests were used for categorical variables. The Kruskal–Wallis test was also used for comparison between more than two groups. Differences were considered statistically significant if *p*-value < 0.05. Statistical analysis was performed by N.E. using SAS 9.4 (SAS Institute, Cary, NC).

## Results

### Patient characteristics

Patients with IgAN with gene expression analyzed by NanoString were mostly Caucasians (76.7%) and females (73.3%), as shown in Table 1. Mean age at biopsy was 44.7 ± 15.3 years. Mean baseline eGFR and proteinuria were 60.9 ± 35.8 ml/min/1.73m^2^, and 3.2 ± 2.9 g/d respectively; most patients (96.5%) had a stage 0 or 1 of fibrosis at diagnosis. At baseline, 60% of patients had chronic kidney disease stage 3 or 4. Treatments were highly variable, but a significant proportion (80%) were on renin–angiotensin–aldosterone (RAA) system inhibitors, and 45% received glucocorticoids at some point in their disease course. Patients with ANCA-associated pauci-immune glomerulonephritis were all Caucasians (100%), with a majority of females (57.1%). Mean age at biopsy was 62.6 ± 9.4 years. At baseline, mean eGFR and proteinuria were 36.6 ± 33 ml/min/1.73m^2^ and 2.4 ± 2.2 g/d, respectively; all patients had stage 0 or 1 of fibrosis at diagnosis. Patients with ANCA-associated pauci-immune glomerulonephritis were significantly older and had more kidney impairment at baseline than patients with IgAN.

**Table 1:** Patient characteristics

Characteristics	ANCA-associated pauci-immune glomerulonephritis (*n* = 7)		IgA nephropathy (*n* = 30)	*P*-value	N missing
Female	4 (57.1)		22 (73.3)	0.1827	
Age at biopsy, yrs	62.6 (9.4)		44.7 (15.3)	0.0096	
BMI at baseline, kg/m^2^	27.2 (4.4)		28.3 (7.0)	0.8442	4
Ethnicity					
Caucasian	7 (100)		23 (76.7)	0.5694	
Afro-American	0		1 (3.3)		
Asian	0		4 (13.3)		
Others	0		2 (6.7)		
EGFR at baseline, ml/min per 1.73 m^2^	36.6 (33.0)		60.9 (35.8)	0.0363	
Proteinuria at baseline, g/d	2.4 (2.2)		3.2 (2.9)	0.4044	
Fibrosis on biopsy, %					
T0	3 (50)		11 (37.9)	0.7975	2
T1	3 (50)		17 (58.6)		
T2	0		1 (3.5)		
Diabetes	1 (14.3)		9 (30)	0.6471	
Hypertension	6 (85.7)		23 (76.7)	1.0000	
Family history of ESKD	0		1 (3.3)	1.0000	
Smoking	4 (57.1)		15 (50)	1.0000	
Medications^a^					
RAAS inhibitors	3 (42.9)		24 (80)	0.0688	
Glucocorticoids	5 (83.3)		9 (45)	0.1696	11
MMF	1 (16.7)		1 (5)	0.4154	11
Ballardie^b^regimen	4 (66.7)		1 (5)	0.0047	11
Cyclophosphamide	3 (50.0)		1 (5)	0.0278	11
Azathioprine	2 (33.3)		1 (5)	0.1231	11
Rituximab	2 (33.3)		0 (0)	0.0462	11

Data are expressed as mean (standard deviation) or *n* (percent)

*ANCA,* antineutrophil cytoplasmic antibody, *IgA* immunoglobulin A, *BMI* body mass index, *EGFR* estimated glomerular filtration rate, *ESKD* end-stage kidney disease, *RAAS* renin–angiotensin–aldosterone system, *MMF* mycophenolate mofetil

^a^Received at any time during the disease course

^b^Glucocorticoids and cyclophosphamide for the initial 3 months, then azathioprine for a minimum of 2 years

The 5-year risk of a 50% decline in eGFR or ESKD after biopsy was variable in IgAN patients: 9, 5, 8, and 6 patients (2 missing) had a 5-year risk of progression of  < 10%, 10%–20%, 21%–40%, and > 40%, respectively. The median follow-up time for the IgAN group was 4.62 years (interquartile range 2.45 years). Among patients with IgAN, 8 patients (26.7%) reached ESKD and 3 (10%) died.

### Gene expression profiles

IgAN patients had a different mRNA expression profile than patients with ANCA-associated pauci-immune glomerulonephritis. *IL-6, INFG,* and *C1QB* were significantly transcribed at higher levels in patients with ANCA-associated pauci-immune glomerulonephritis (Table 2). *AGTR1* was more expressed in patients with IgAN compared to ANCA controls (*p* = 0.05).

**Table 2:** Distribution of gene expression between IgAN and ANCA patients

Genes	ANCA		IgAN		*P*-value
	*n* = 7	SD	*n* = 30	SD	
*AGTR1*	64.7	28.7	162.2	119.8	0.0470
*AGTR2*	3.2	2.0	9.3	12.6	0.1433
*C1QA*	42.3	36.5	19.8	13.8	0.0795
*C1QB*	1091.0	919.7	395.9	305.3	0.0180
*C3*	402.2	554.7	177.9	162.5	0.3356
*CD71*	103.8	45.5	154.5	103.7	0.3787
*CD89S*	3.0	1.9	3.3	2.2	0.9144
*CXCL1*	175.3	141.4	99.8	73.0	0.3355
*ICAM1*	197.7	147.5	127.8	83.1	0.2865
*IFNG*	6.5	7.7	1.9	2.0	0.0175
*IL10*	3.2	2.6	2.4	1.7	0.5118
*IL13*	4.8	2.6	3.5	2.6	0.2276
*IL2*	1.8	1.0	1.4	0.7	0.2471
*IL4*	1.7	1.2	1.1	0.4	0.0703
*IL5*	4.0	2.8	2.7	1.5	0.3690
*IL6*	9.7	5.3	3.4	3.1	0.0076
*IL8*	69.3	82.9	26.8	22.3	0.1896
*MIF*	1268.3	599.5	1384.8	631.1	0.7842
*PTEN*	390.3	237.1	470.0	241.6	0.5149
*TGFB1*	337.8	210.9	302.1	130.8	0.8168
*TGM2*	373.5	232.3	259.4	143.4	0.2345
*TNF*	41.7	37.4	27.8	19.3	0.5701
*TNFR1B*	434.0	231.9	447.7	251.1	0.8496
*TNFRSF1B*	140.2	96.1	102.9	50.8	0.2682
*TNFSF13*	82.5	42.8	75.1	29.3	0.5699
*BAD*	101.5	33.4	111.4	42.0	0.5013
*BAX*	321.0	168.4	311.0	160.5	0.8496
*C2*	125.2	115.3	73.1	68.4	0.3153
*CASPASE3*	111.2	83.3	93.2	54.7	0.8331
*ACTB*	7481.0	4373.3	7052.5	3307.5	0.8496
*GAPDH*	4209.8	1800.4	4779.9	3293.8	1.0000
*HPRT1*	235.5	117.2	238.7	135.1	0.8496
*LDHA*	1352.8	585.5	1346.0	815.7	0.9496

*ANCA* antineutrophil cytoplasmic antoantibody, *IgAN* immunoglobulin A nephropathy

The distribution of biomarkers in function of the IgA nephropathy prediction tool score range was analyzed. Markers known as *CIQA*, *ICAM1*, *TNF,* and *TNFRSF1B* did not show a statistically significant distribution, but their distribution difference was near the significance level (Table 3). As shown in Table 4, *CIQB and TNFRSF1B* expressions were higher among patients with an IgA prediction tool score range of 10–20% compared to a score < 10% (*p*-value = 0.01342 and *p* = 0.03337, respectively). *C3* expression was also higher in patients with a score range of 10–20% (vs. with a score < 10%) with a *p*-value of 0.04911.

**Table 3:** Distribution of gene expression by Risk-Prediction Tool score ranges

Genes	Score < 10%		Score 10–20%		Score 20–40%		Score 40%		*P*-value*
	*n* = 9	%	*n* = 5	%	*n* = 8	%	*n* = 6	%	
*AGTR1*	154.56	120.2	251.6	133.5	144.38	97.3	141.83	148.1	0.3845
*AGTR2*	6.78	6.4	4.4	4.2	13.88	21.9	10.67	9.7	0.7307
*C1QA*	14.11	8.9	36.4	17.0	18.5	10.7	17	13.8	0.0654
*C1QB*	255.33	156.5	736.8	565.1	390.63	148.2	355.5	212.2	0.1057
*C3*	116.11	124.2	318.4	205.0	159.63	118.2	150	143.2	0.1589
*CD71*	167.56	109.8	212	132.1	145.75	91.3	110.67	97.9	0.4605
*CD89S*	3.56	2.8	2.8	1.1	3.25	2.6	3.5	2.1	0.9565
*CXCL1*	73	68.0	113.2	47.8	111	78.9	116	96.8	0.6398
*ICAM1*	99	66.6	196.6	34.9	137.63	105.7	98.17	85.1	0.0838
*IFNG*	1.33	0.7	3.4	4.3	2	1.6	1.5	0.8	0.7648
*IL10*	2	1.1	2.6	1.1	3	2.0	1.33	0.5	0.1614
*IL13*	3.67	2.5	6	3.5	2.88	2.2	2.17	1.2	0.157
*IL2*	1.11	0.3	1.6	0.6	1.5	0.9	1.33	0.8	0.3551
*IL4*	1.22	0.7	1.2	0.5	1	0.0	1	0.0	0.5018
*IL5*	3	1.1	3.2	2.5	2.63	1.7	2.17	1.2	0.6339
*IL6*	3.67	3.3	5	3.2	2.13	1.4	2.33	2.0	0.2006
*IL8*	22.78	22.5	29.8	15.5	35.88	30.5	17.33	12.7	0.4965
*MIF*	1409.22	674.8	1796	439.1	1466.88	654.6	958.67	590.1	0.2334
*PTEN*	413.11	216.8	620.2	244.4	493.5	208.4	408.83	315.3	0.487
*TGFB1*	218.33	109.1	371.4	70.8	346.63	118.5	304.83	173.9	0.1202
*TGM2*	196.22	149.9	318	63.3	328.38	128.8	209.5	158.1	0.2137
*TNF*	17.33	10.7	40.8	16.2	32.88	19.6	26.83	27.7	0.0873
*TNFR1B*	394.33	201.7	682.2	285.1	440.25	220.9	334.17	252.8	0.2078
*TNFRSF1B*	70.78	36.8	140.8	47.0	116.63	36.0	99.67	67.5	0.0602
*TNFSF13*	62.33	31.0	82.4	8.2	90.75	30.0	66	35.0	0.2166
*BAD*	106.11	49.4	121.6	21.7	120	42.7	103.67	48.9	0.8908
*BAX*	274.78	137.4	424.8	135.6	319.63	155.6	251	181.1	0.2558
*C2*	79.11	88.4	91.4	68.0	76.88	70.3	41.5	25.0	0.5412
*CASPASE3*	72.56	39.3	134.4	58.9	102.38	46.3	78.67	70.8	0.1902

*Kruskal–Wallis test for comparison between more than two groups. Two patients have the prediction tools missing

**Table 4:** Mean difference of gene expression between Risk-Prediction Tool score groups < 10% and 10–20%

Genes	Score < 10%		Score 10–20%		Score < 10 vs 10–20%		*P*-value
	*n* = 9	SD	*n* = 5	SD	Mean diff	SE	
*AGTR1*	154.56	120.2	251.6	133.5	97.04	68.6	0.38664
*AGTR2*	6.78	6.4	4.4	4.2	-2.38	7.4	0.97968
*C1QB*	255.33	156.5	736.8	565.1	481.47	155.0	0.01342
*C3*	116.11	124.2	318.4	205.0	202.29	79.9	0.04911
*CD71*	167.56	109.8	212	132.1	44.44	59.5	0.81152
*CD89S*	3.56	2.8	2.8	1.1	− 0.76	1.3	0.90161
*CXCL1*	73	68.0	113.2	47.8	40.20	42.1	0.67982
*ICAM1*	99	66.6	196.6	34.9	97.60	44.8	0.10232
*IFNG*	1.33	0.7	3.4	4.3	2.07	1.1	0.20489
*IL10*	2	1.1	2.6	1.1	0.60	0.8	0.78642
*IL13*	3.67	2.5	6	3.5	2.33	1.4	0.23824
*IL2*	1.11	0.3	1.6	0.6	0.49	0.4	0.47247
*IL4*	1.22	0.7	1.2	0.5	− 0.02	0.2	0.99947
*IL5*	3	1.1	3.2	2.5	0.20	0.9	0.99288
*IL6*	3.67	3.3	5	3.2	1.33	1.4	0.69613
*IL8*	22.78	22.5	29.8	15.5	7.02	12.7	0.90954
*MIF*	1409.22	674.8	1796	439.1	386.78	344.5	0.5679
*PTEN*	413.11	216.8	620.2	244.4	207.09	135.5	0.32601
*TGFB1*	218.33	109.1	371.4	70.8	153.07	68.8	0.09321
*TGM2*	196.22	149.9	318	63.3	121.78	75.2	0.28267
*TNF*	17.33	10.7	40.8	16.2	23.47	10.5	0.09101
*TNFR1B*	394.33	201.7	682.2	285.1	287.87	130.4	0.09666
*TNFRSF1B*	70.78	36.8	140.8	47.0	70.02	25.8	0.03337
*TNFSF13*	62.33	31.0	82.4	8.2	20.07	16.3	0.495
*BAD*	106.11	49.4	121.6	21.7	15.49	24.5	0.87348
*BAX*	274.78	137.4	424,8	135.6	150.02	85.0	0.22153
*C2*	79.11	88.4	91.4	68.0	12.29	39.2	0.98119
*CASPASE3*	72.56	39.3	134.4	58.9	61.84	29.3	0.11736
*ACTB*	5964.11	2891.5	9102.2	1809.4	3138.09	1843.7	0.24625
*GAPDH*	5045.22	2969.0	7142.6	4504.5	2097.38	1797.5	0.53886
*HPRT1*	236.78	126.2	311.8	112.5	75.02	77.3	0.66952
*LDHA*	1259.22	714.4	1744.2	626.0	484.98	470.8	0.62995

### Immunofluorescence

**Fig. 1:**
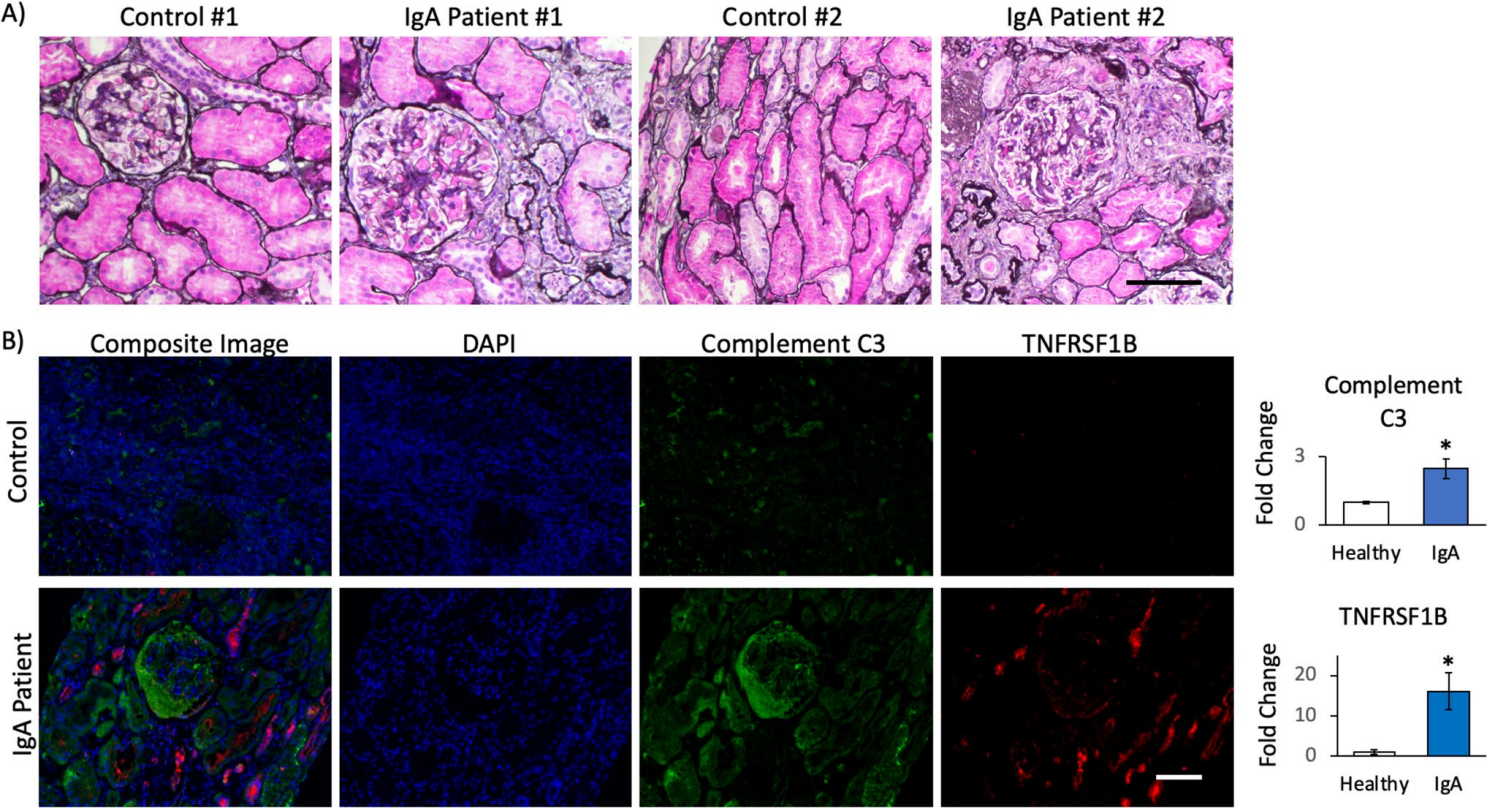
Kidney biopsies from IgA patients have increased fibrosis and immune markers. **A** Light microscopy of kidney biopsies stained for H&E shows increased fibrosis in IgA nephropathy patients compared to controls. **B** Immunofluorescence staining for Complement C3 and TNFRSF1B in IgA nephropathy patients compared to controls. Fold change of immunofluorescence, as represented in a bar graph, was performed using *n* = 6 IgA patients and *n* = 3 controls. *P < 0.05 determined by *t*-test. Scale bars: 100 μm

To ensure the accuracy and reliability of our findings obtained through NanoString mRNA profiling, we devised a validation strategy that involved the examination of protein expression levels of candidate genes. To conduct our protein expression analyses, we selected *Complement C3* as a marker of mild to moderate risk of ESKD progression with relatively minimal fibrosis on kidney biopsy, and *TNFRSF1B* to serve as a marker expressed during stage 2 fibrosis. To this end, we obtained biopsies from patients diagnosed with IgA nephropathy that had high degree of fibrosis compared to the controls (Fig. 1A). Our findings revealed that both target genes identified in gene expression analysis (*C3* and *TNFRSF1B*) exhibited a significant upregulation of protein expression in the biopsies obtained from patients with IgA nephropathy when compared to control tissue (Fig. 1B). Importantly, the expression patterns of Complement C3 and TNFRSF1B were distinct, further underscoring the significance of our observations. Complement C3 was found to be distributed around the tubular epithelial cells and glomerulus, with a modest signal, suggesting a possible role in the regulation of inflammation and immune responses in such regions. On the other hand, the signal for TNFRSF1B was more pronounced and concentrated in distinct regions that differed from those observed for Complement C3, highlighting its potential role in cellular proliferation and differentiation. Overall, our findings validate our NanoString mRNA profiling by examining protein expression levels of two candidate genes, *C3* and *TNFRSF1B*, in IgAN patients and controls.

### NanoString identified mRNA expressed targets as predictors of fibrosis

**Fig. 2:**
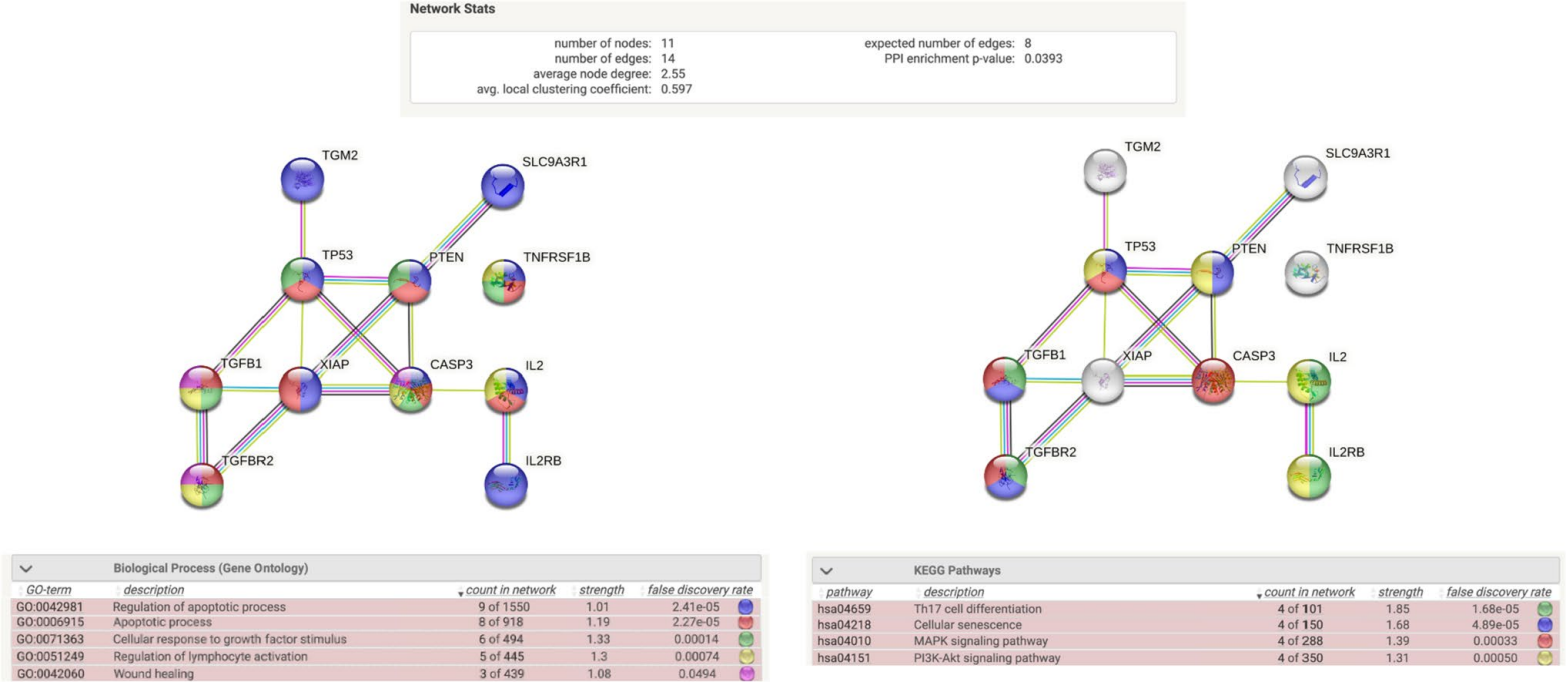
Interaction analysis of differentially expressed genes from kidneys of IgA patients. Using the STRING database, we determined the predicted interactome, biological processes (left), and signaling pathways (right) from significantly upregulated genes within IgA kidney biopsies compared to controls. Color codes of nodes are based on the annotation term for each “Biological Function” or “KEGG Pathway,” and interaction edges are based on a minimal confidence of 0.7 using sources of text mining, experiments, databases, and co-expressions

Fibroblasts are responsible for producing and depositing extracellular matrix components and may be stimulated by persistent inflammation and immunological responses, such as those during IgAN. The excessive production and accumulation of collagen and other matrix proteins lead to scar tissue formation, known as fibrosis. Therefore, we analyzed our IgAN patient samples for fibrosis formation and compared the severity of fibrosis with the NanoString mRNA transcript expression of our targets. From our histological slides, we observed fibrosis formation in IgAN patients that resulted in structural changes within the kidney, including thickening of the glomerular basement membrane, obliteration of the glomerular capillaries, and an increase in the volume of the interstitial compartment (Fig. 1A). From our NanoString mRNA expression profiles, we observed a significant increase in *PTEN*, *CASPASE 3*, *TGM2*, *TGFB1*, *IL2*, and *TNFRSF1B* from IgAN patients with level 2 fibrosis compared to those with none (Table 5). To gain insights into such significantly upregulated mRNA from IgA fibrotic patients, we compiled them into the STRING platform (https://string-db.org/) to analyze any potential biological processes and interactions in the form of an interaction network. Set at a high confidence of 0.7, our identified “fibrotic” genes did not show any strong correlation among themselves. However, when adding 5 more relevant nodes to the network, *IL2RB*, *SLC9A3R1*, *TP53*, *TGFBR2,* and *XIAP*, we were able to establish a more complete network of associating genes, with a total of 11 nodes and 14 edges (Fig. 2). The protein–protein interaction networks (PPI) enrichment p-value of 0.0393 indicates that the nodes are not random and that the observed number of edges is significant. When studying the various descriptions in the “Biology Process” category, we identified that such genes are primarily involved in apoptotic pathways, regulation of apoptosis, wound healing, response to growth factors, and regulation of lymphocyte activation, while the meaningful “Pathways” included Th17 cell differentiation, cellular senescence, MAPK signaling pathway, and PI3K-Akt signaling pathway. Therefore, our study identified several upregulated mRNA transcripts that were upregulated during the development of fibrosis and may be considered fibrotic markers within IgAN patients.

**Table 5:** Gene expression in biopsies of patients with stage 0 versus stage 2 fibrosis

Genes	Detected RNA transcripts				Ratio	*P*-value
	Stage 0	SD	Stage 2	SD		
*PTEN*	421.24	92.77	554.67	84.27	− 1.32	0.0084
*CASPASE 3*	78.76	18.00	109.64	28.43	− 1.39	0.0106
*TGM2*	191.84	121.35	353.69	165.28	− 1.82	0.0413
*TGFB1*	235.89	146.21	456.21	128.66	− 1.93	0.0087
*IL2*	1.29	0.54	2.64	2.41	− 2.05	0.0247
*TNFRSF1B*	75.83	52.50	158.20	44.83	− 2.09	0.0097

*RNA* ribonucleic acid, *SD* standard deviation

## Discussion

Our pilot study aimed to validate the use of the NanoString technology in identifying potential inflammatory targets at different stages of IgAN disease progression. IgAN patients appear to have a distinct inflammatory profile from other inflammatory glomerulonephritis. We were able to identify *C3* and *TNFRSF1B* as potential biomarkers of early inflammation in patients with a moderately increased risk of disease progression. When compared to controls and using an immunofluorescence technique, protein expression for those two markers was more pronounced, with distinct signal patterns. Those biomarkers might be implicated in the development of fibrosis when studying potential biological processes and interactions in the form of an interaction network.

Interestingly, the tumor necrosis factor (TNF) receptor type 2 (TNFR2, also known as TNFRSF1B) is expressed both on activated effector T cells and regulatory cells, such as regulatory T cells (Tregs) and myeloid-derived suppressor cells. As such, it has contradictory pro-inflammation and anti-inflammation properties. Indeed, TNFRSF1B deficiency could, in some cases, worsen autoimmune diseases, and TNFRSF1B agonists might have opposite effects (reviewed in [9]). It has been suggested that patients with IgAN have a decreased Treg number [10–12] and suppressive potential [13]. However, we do not know if the TNFRSF1B expression we detected in the kidney was on Tregs, activated conventional T cells, or other cell types. In an epigenome-wide association study of the Framingham’s cohort, soluble TNFRSF1B (sTNFRSF1B) levels in the blood were associated with differentially methylated loci in active regulatory regions of genes previously identified in GWAS studies of IgAN patients, suggesting a possible role in the disease [14]. TNFRSF1B mRNA and protein levels is also elevated in IgAN patients [15], correlates with progression [16, 17], and could decrease with steroids [18]. However, the *TNFRSF1B* expression in the kidney has never been studied and does not appear to be expressed in IgA-dominant infection-related glomerulonephritis [19]. Complement C3 has a crucial role in IgAN, colocalizing with IgA in over 90% of patients [20]. The direct activation of C3 by IgA1-containing complexes forms a vital aspect of the immune pathogenesis in IgAN [21]. This observation aligns with and reinforces our study findings. Furthermore, our ICC co-staining of C3 and TNFRSF1B with fibrosis implies that the early RNA expression of *C3* could serve as a prognostic marker of fibrosis, while *TNFRSF1B* RNA expression associates with the onset of fibrosis.

Fibrosis is a common phenomenon of IgAN, which we confirmed within our study using Jones’ staining. In order to find an association of our significantly upregulated genes with the onset of fibrosis, we compared the expression of such genes between IgAN patients with grade 2 fibrosis to those without fibrosis. As a result, we identified 6 genes that were significantly associated with fibrosis: *PTEN*, *CASPASE 3*, *TGM2*, *TGFB1*, *IL2*, and *TNFRSF1B*. Interestingly, such genes were predicted to be associated with the biological functions of wound healing, apoptosis, cellular response to growth factors, and regulation of immune systems.

We also predicted various relevant pathways belonging to our IgA nephropathic genes that are significantly upregulated during fibrotic tissue formation. These include Th17 cell differentiation, cellular senescence, and the MAPK and PI3K-Akt signaling pathways. It has been shown that the abnormal humoral immunity observed in IgAN may be mediated by the differentiation of T helper 17 cells [22] and kidney senescence is well documented to occur after injury [23]. It has been observed that Akt activation is increased in experimental tubulointerstitial fibrosis [24, 25]. Akt activation serves as a crucial component in multiple signaling pathways associated with kidney damage. Consequently, there is a belief that Akt plays a significant role in renal fibrosis. The MAPK pathway plays a pivotal role in regulating inflammatory and fibrotic responses within the kidney [26, 27]. Notably, one significant outcome of MAPK pathway activation is the induction of extracellular matrix protein synthesis, particularly collagen. The excessive deposition of collagen and other extracellular matrix components contributes to the progressive accumulation of fibrotic tissue in the kidney. Thus, the MAPK pathway serves as a key mediator in the development and progression of renal fibrosis by modulating various cellular and molecular events involved in fibrotic remodeling. These findings support the potential involvement of these pathways in driving the fibrotic process and provide valuable directions for further exploration.

Unfortunately, we failed to identify some biomarkers associated with a higher score at the IgA Nephropathy Prediction tool. Some biomarkers, known as *C1QA, ICAM1, TNF,* and *TNFRSF1B*, showed a distribution in function of the IgA prediction tool score range near a statistically significant level. Both *ICAM1* and *TNF* were close to being associated with a score range of over 20% at the IgAN prediction score. High levels of *ICAM-1* mRNA were associated with advanced histologic changes and lower kidney function in previous studies on IgAN [28]. TNF-alpha is also suspected to play an essential part in the pathogenesis of IgAN. Previous studies established a relation between high serum levels of TNF-a in patients with IgAN and MEST-C scores, higher proteinuria, and lower kidney function [29].

Our present study has some limitations. First, the small number of cases reduced the power of our study and reduced the chances of finding statistically significant differences in the distribution of the biomarkers analysis with the NanoString technology for the clinically relevant endpoints we tried to focus on. However, the purpose of the study was not to elaborate a prediction model using inflammatory biomarkers but to validate NanoString technology as a potential tool to delineate inflammatory processes in IgAN patients with ongoing kidney injury. Another limitation of our study is the limited number of candidate genes examined, mainly because of technical reasons. Nonetheless, we were able to focus on the main targets already described in the literature as involved in IgAN physiopathology.

In conclusion, our study validates the use of the NanoString technology as a promising tool for gene expression analysis in IgAN. Utilizing this innovative platform, we could simultaneously study multiple inflammatory biomarkers involved in IgAN and identify their activation patterns. We also validated the activation of genes known to contribute to kidney fibrosis, shedding light on the underlying mechanisms of disease progression. Although these findings confirm the usefulness of gene expression profiles in providing valuable tools for diagnosis and monitoring IgAN, further research is necessary to elucidate the clinical utility of gene expression analysis and its integration into predictive models and treatment strategies for IgAN. Further exploration of gene expression profiles holds promise for a better comprehension of disease and the development of targeted therapies to mitigate kidney fibrosis in IgAN patients.

## Data Availability

For access to original deidentified data, please contact louisphilippe. laurin@umontreal.ca.
